# Renal denervation for hypertension management: convergence and divergence across international guidelines

**DOI:** 10.1016/j.ijcrp.2026.200618

**Published:** 2026-03-03

**Authors:** Dominique Stephan, Emma Morisot, Elena-Mihaela Cordeanu

**Affiliations:** Hypertension and Vascular Diseases, ESH Blood Pressure Clinic, University Hospital of Strasbourg, France

**Keywords:** Renal denervation, Resistant hypertension, Guidelines

## Abstract

**Background:**

Renal denervation (RDN) has emerged as an interventional option for hypertension management. Three major international guidelines have recently addressed RDN indications and recommendations.

**Objectives:**

To compare and synthesize the positions of the European Society of Cardiology (ESC) 2024, European Society of Hypertension (ESH) 2023, and American College of Cardiology/American Heart Association (ACC/AHA) 2025 guidelines regarding RDN.

**Methods:**

Structured comparative analysis of guideline recommendations using predefined domains: patient selection criteria, recommendation strength and level of evidence, procedural requirements, and clinical positioning. Each guideline was independently reviewed by two authors, with discrepancies resolved by consensus.

**Conclusions:**

All three guidelines converge on four major indications: uncontrolled hypertension, resistant hypertension, drug intolerance, and patient preference. Key divergences relate to the strength of recommendations and specific patient selection criteria. Although differences in recommendation classes (ACC/AHA 2b vs. ESC/ESH IIb) appear discordant, they largely reflect structural variations in grading frameworks rather than genuine differences in evidence appraisal. The modest expected blood pressure reduction (4–6 mmHg ambulatory systolic), equivalent to approximately one antihypertensive medication, translates into an estimated 10% reduction in major cardiovascular events, though individual responses vary considerably. RDN represents a complementary therapeutic option requiring multidisciplinary evaluation and shared decision-making.

## 1 Introduction

Catheter-based renal denervation (RDN) targets the sympathetic nervous system overactivity implicated in hypertension pathophysiology. Following the initially disappointing results of SYMPLICITY HTN-3, second-generation devices using radiofrequency or ultrasound energy have demonstrated consistent blood pressure (BP) reductions in sham-controlled trials [1,2]. Three major international societies have recently published or updated their positions on RDN: the ESH in 2023 [3], the ESC in 2024 [4], and the ACC/AHA in 2025 [5]. This review analyzes the convergent and divergent aspects of these recommendations.

## 2 Methods

The three guidelines were selected as the most recent comprehensive hypertension management documents issued by the major international societies with established positions on RDN. A structured comparative analysis was conducted using predefined domains: (i) patient selection criteria, including indications, blood pressure thresholds, and renal function requirements; (ii) recommendation strength and level of evidence, reported using each society's original grading system; (iii) procedural requirements, including centre expertise and multidisciplinary evaluation; and (iv) clinical positioning, specifically whether RDN was framed as complementary or stand-alone therapy. Each guideline was independently reviewed by two authors (DS and EM), with extracted data cross-checked for accuracy. Discrepancies in interpretation were resolved by consensus with the third author (EMC). Given the non-equivalent grading frameworks employed by the ACC/AHA and ESC/ESH, recommendation classes were reported in their original nomenclature and were not directly equated across systems. A correspondence table (Table 2) was constructed to facilitate comparison.

**Table 1:** Comparative analysis of international guideline recommendations on renal denervation.

Domain	ACC/AHA 2025	ESC 2024	ESH 2023
**Indications**			
Uncontrolled HTN	✓	✓	✓
Resistant HTN	✓	✓	✓
Drug intolerance	✓	✓	✓
Patient preference	✓	✓	✓
Non-resistant HTN (<3 drugs)	–	✓ (IIb/A)	–
**BP thresholds**			
Office SBP	140–180 mmHg	Not specified	Not specified
Office DBP	≥90 mmHg	Not specified	Not specified
**Renal function**			
eGFR threshold	≥40 mL/min/1.73 m^2^	≥40 mL/min/1.73 m^2^	>40 mL/min/1.73 m^2^
**Recommendation**			
Class/Level	2b/B-R	IIb/B (resistant) IIb/A (<3 drugs)	II/B
First-line therapy	Not addressed	Class III (not recommended)	Not addressed
**Procedural requirements**			
Specialized centre	Class I, B-NR	✓ (medium-to-high volume)	✓ (Class I)
Multidisciplinary team	Class I, B-NR	✓	✓ (Class I)
Shared decision-making	Class I, C-EO	✓	✓ (Class I)
**Expected efficacy (ambulatory SBP)**			
Medication-free	4–6 mmHg	≈4 mmHg (ABPM)	Comparable figures
On medications/resistant	3–5 mmHg (variable)	≈6 mmHg (office)	Individual trial data
Complementary role (not curative)	✓	✓	✓

ACC/AHA: American College of Cardiology/American Heart Association; ABPM: ambulatory blood pressure monitoring; BP: blood pressure; DBP: diastolic blood pressure; eGFR: estimated glomerular filtration rate; ESC: European Society of Cardiology; ESH: European Society of Hypertension; HTN: hypertension; SBP: systolic blood pressure. ✓ = recommended/addressed; – = not addressed.

**Table 2:** Summary of recommendation classes and levels of evidence for renal denervation.

Guideline	Class/Level	Key Requirements
ACC/AHA 2025	2b/B-R	SBP 140–180 mmHg, DBP ≥90 mmHg; eGFR ≥40; multidisciplinary team (Class I); shared decision-making (Class I)
ESC 2024	IIb/B (resistant HTN) IIb/A (<3 drugs)	Medium-to-high volume center; eGFR ≥40; shared risk-benefit discussion; NOT recommended as first-line (Class III)
ESH 2023	II/B	eGFR >40; specialized centers (Class I); shared decision-making (Class I)

DBP: diastolic blood pressure; eGFR: estimated glomerular filtration rate (mL/min/1.73 m^2^); HTN: hypertension; SBP: systolic blood pressure. ACC/AHA classification: Class 2b = may be reasonable (benefit ≥ risk, with less certainty); Level B-R = moderate evidence from Randomized trials; Level B-NR = moderate evidence from Non-Randomized studies; Level C-EO = consensus Expert Opinion. ESC/ESH classification: Class I = recommended; Class IIa = should be considered; Class IIb = may be considered; Class III = not recommended. Level A = multiple randomized trials or meta-analyses; Level B = single randomized trial or large non-randomized studies; Level C = expert consensus.

## 3 Points of convergence

### 3.1 Patient selection criteria

All three guidelines agree on four fundamental indications for considering RDN (Table 1): uncontrolled hypertension despite pharmacotherapy, resistant hypertension confirmed by ambulatory BP monitoring (ABPM), intolerance to antihypertensive medications with significant side effects, and patient preference for an interventional approach. Renal function threshold. A consistent criterion across guidelines is preserved renal function, with an estimated glomerular filtration rate (eGFR) ≥40 mL/min/1.73 m^2^ required for RDN consideration. The ESC and ESH explicitly contraindicate RDN in patients with moderate-to-severe renal impairment until further evidence emerges.

### 3.2 Procedural requirements

All societies emphasize that RDN should be performed exclusively in experienced, specialized, medium-to-high volume centers with established multidisciplinary teams. The ACC/AHA assigns this recommendation a Class I, Level B-NR, requiring evaluation by a multidisciplinary team with expertise in resistant hypertension and RDN. Shared decision-making. A cornerstone recommendation across all guidelines is the mandatory shared decision-making process. Patients must be fully informed about the benefits, limitations, procedural risks, and the modest expected BP reduction. The ACC/AHA specifically mandates discussion of benefits compared with continuing medical therapy (Class I, Level C-EO).

### 3.3 Complementary role

None of the guidelines position RDN as a curative therapy or full replacement for antihypertensive drugs. All emphasize that RDN is an adjunct treatment to lifestyle modifications and pharmacotherapy, not a first-line intervention.

## 4 Points of divergence

### 4.1 Strength of recommendations

The guidelines differ in their level of endorsement. The ESC provides Class IIb recommendations (may be considered), reflecting concerns about the lack of cardiovascular outcomes trials. Similarly, the ESH assigns Class II recommendations. The ACC/AHA takes a more cautious stance with a Class 2b, Level B-R recommendation, explicitly noting that broader FDA-approved indications exist but that modest BP-lowering effects and absence of outcome data limit the role of RDN. Interpretation of grading framework differences. Despite the apparent divergence in nomenclature, the ACC/AHA Class 2b (“may be reasonable, benefit ≥ risk with less certainty”) and the ESC/ESH Class IIb (“may be considered”) convey a similar degree of clinical uncertainty. This suggests that the cross-guideline discrepancy in recommendation class is largely structural, reflecting the inherent differences between the American and European grading systems, rather than a substantive disagreement on the clinical role of RDN. However, genuine divergences in evidence appraisal do exist. Notably, the ESC assigns Level A evidence to RDN in non-resistant hypertension (<3 drugs), reflecting the weight given to sham-controlled trial data in medication-free or low-medication populations (RADIANCE-HTN SOLO, SPYRAL HTN-OFF MED), whereas the ACC/AHA assigns Level B-R to the broader recommendation. This distinction represents a differential weighting of the same trial evidence base rather than access to different datasets.

### 4.2 Quantification of expected efficacy

The ACC/AHA guidelines provide specific efficacy data: in medication-free patients, RDN induces 24-h ambulatory systolic BP reduction of 4 to 6 mmHg over 2–3 months. In patients on 2–5 antihypertensive agents or with resistant hypertension, results are variable, with some trials showing 3–5 mmHg reduction versus sham while others failed primary endpoints. The ESC cites meta-analyses showing approximately 6 mmHg office BP and 4 mmHg ambulatory BP reduction. The ESH provides comparable figures from individual trials. Blood pressure thresholds. The ACC/AHA specifies BP entry criteria for RDN consideration: office systolic BP 140–180 mmHg and diastolic BP ≥ 90 mmHg. The European guidelines define resistant hypertension but do not specify upper BP limits for RDN eligibility.

### 4.3 Non-resistant hypertension

The ESC uniquely provides a Class IIb, Level A recommendation for patients with increased cardiovascular risk and uncontrolled hypertension on fewer than three drugs who express a preference for RDN. This extends beyond traditional resistant hypertension populations, though the higher level of evidence reflects the stronger trial data in this population.

### 4.4 Emphasis on limitations

The ACC/AHA guidelines explicitly caution that RDN should not be considered curative or a replacement for medication, noting the relatively short follow-up duration and absence of cardiovascular outcome trials. The ESC provides a Class III (not recommended) statement against RDN as first-line therapy, with specific concern that the “always-on” nature of RDN could be problematic if late complications emerge.

### 4.5 Synthesis and clinical implications

The convergence across international guidelines (Table 1) establishes RDN as a recognized complementary option for selected hypertensive patients. The shared emphasis on specialized centers, multidisciplinary evaluation, and patient-centered decision-making reflects a measured approach to implementation. Divergences primarily concern the degree of enthusiasm, reflecting different institutional philosophies and weighting of the evidence. The ACC/AHA adopts the most conservative position, explicitly acknowledging FDA approvals while tempering expectations. The ESC provides the most detailed analysis of concerns, including cost-effectiveness questions and workflow considerations. The ESH offers practical guidance focusing on patient selection and procedural quality. Although the mean BP reduction achieved with RDN appears modest (4–6 mmHg ambulatory systolic), this magnitude of effect carries meaningful clinical significance. Epidemiological data from large-scale meta-analyses indicate that a sustained 5 mmHg reduction in systolic BP is associated with an approximately 10% reduction in major cardiovascular events, including stroke and myocardial infarction [6]. Importantly, the population-level mean effect conceals substantial inter-individual variability: while some patients experience negligible BP changes, others achieve clinically significant reductions exceeding 15–20 mmHg. Post-hoc analyses of sham-controlled trials have identified potential predictors of favourable response, including higher baseline sympathetic activity, preserved renal function, and absence of isolated diastolic hypertension, although these predictors require prospective validation [7]. This heterogeneity of response underscores the importance of individualized patient counselling during the shared decision-making process. These guideline divergences carry practical implications that differ between the US and European clinical settings. In the United States, the ACC/AHA's explicit BP entry criteria (systolic 140–180 mmHg, diastolic ≥90 mmHg) and the regulatory availability of FDA-cleared devices (Symplicity Spyral™, Paradise™) provide a well-defined clinical pathway with clear eligibility boundaries. In Europe, the absence of specific BP thresholds in the ESC and ESH guidelines affords greater clinical flexibility but may result in heterogeneity in patient selection across centers. Furthermore, the ESC's unique recommendation for RDN in patients on fewer than three antihypertensive agents with elevated cardiovascular risk extends the eligible population beyond traditional resistant hypertension, a nuance not reflected in the American guidelines. These differences are likely to influence referral patterns, resource allocation, training requirements, and reimbursement policies across healthcare systems. The universal requirement for specialized high-volume centers may create access disparities, particularly in healthcare systems with limited interventional cardiology or radiology capacity.

In clinical practice, the modest BP reduction (equivalent to approximately one antihypertensive medication) positions RDN for patients where medication adherence is problematic, intolerable side effects limit pharmacotherapy, or patient preference strongly favors a procedural intervention. The requirement for preserved renal function excludes patients with advanced chronic kidney disease, a population with substantial unmet needs. The absence of cardiovascular outcome trials remains the critical evidence gap preventing stronger recommendations. Until such data emerge, RDN will remain a Class II/IIb option, appropriate for carefully selected patients within a framework of shared decision-making and specialized care.

## 5 Conclusion

The ESC 2024, ESH 2023, and ACC/AHA 2025 guidelines demonstrate substantial convergence on RDN positioning as a safe, complementary option for selected patients with uncontrolled or resistant hypertension, drug intolerance, or strong patient preference. Although differences in recommendation class nomenclature may suggest disagreement, the underlying clinical message is largely concordant: RDN may be considered in carefully selected patients after thorough multidisciplinary evaluation. The modest mean BP reduction of 4–6 mmHg, while clinically meaningful at the population level, is accompanied by substantial inter-individual variability, reinforcing the need for realistic patient expectations and individualized counselling. Divergences in recommendation strength reflect the current evidence limitations, particularly the absence of cardiovascular outcome data. Implementation requires specialized centers, multidisciplinary teams, and thorough informed consent processes. Future outcome trials will be essential to refine these recommendations and to determine whether predictors of individual response can improve patient selection.

## CRediT authorship contribution statement

**Dominique Stephan:** Writing – review & editing, Writing – original draft, Validation, Supervision, Methodology, Formal analysis, Data curation, Conceptualization. **Emma Morisot:** Writing – original draft, Validation, Formal analysis, Data curation. **Elena-Mihaela Cordeanu:** Writing – original draft, Validation, Methodology, Formal analysis, Data curation, Conceptualization.

## Declaration of generative AI and AI-assisted technologies in the manuscript preparation process

During the preparation of this work, the authors used Claude (Anthropic) and ChatGPT (OpenAI) for the following purposes: (1) English language polishing and grammatical revision; (2) formatting of tables and references; and (3) summarisation of lengthy guideline passages to facilitate cross-comparison. The intellectual core of the work, the selection of comparison domains, the identification of convergences and divergences, the interpretation of recommendation classes, and the formulation of clinical implications, was performed entirely by the authors. All AI-generated output was critically reviewed, verified against the original guideline documents, and edited as needed. The authors take full responsibility for the content of the published article.
